# Pancreatic Cancer Action Network's SPARK: A Cloud-Based Patient Health Data and Analytics Platform for Pancreatic Cancer

**DOI:** 10.1200/CCI.23.00119

**Published:** 2024-01-02

**Authors:** Kawther Abdilleh, Omar Khalid, Dennis Ladnier, Wenshuai Wan, Sara Seepo, Garrett Rupp, Valentin Corelj, Zelia F. Worman, Divya Sain, Jack DiGiovanna, Bruce Press, Satty Chandrashekhar, Eric Collisson, Karen Y. Cui, Anirban Maitra, Paul A. Rejto, Kevin P. White, Lynn Matrisian, Sudheer Doss

**Affiliations:** ^1^ Pancreatic Cancer Action Network, Manhattan Beach, CA; ^2^ Tempus, Chicago, IL; ^3^ Velsera, Charlestown, MA; ^4^ ZS, Princeton, NJ; ^5^ Boston Consulting Group, New York, NY; ^6^ University of California San Francisco Helen Diller Family Comprehensive Cancer Center, San Francisco, CA; ^7^ Astra Zeneca, Rockville, MD; ^8^ The University of Texas MD Anderson Cancer Center, Houston, TX; ^9^ Pfizer, La Jolla, CA; ^10^ National University of Singapore, Singapore, Singapore

## Abstract

**PURPOSE:**

Pancreatic cancer currently holds the position of third deadliest cancer in the United States and the 5-year survival rate is among the lowest for major cancers at just 12%. Thus, continued research efforts to better understand the clinical and molecular underpinnings of pancreatic cancer are critical to developing both early detection methodologies as well as improved therapeutic options. This study introduces Pancreatic Cancer Action Network's (PanCAN's) SPARK, a cloud-based data and analytics platform that integrates patient health data from the PanCAN's research initiatives and aims to accelerate pancreatic cancer research by making real-world patient health data and analysis tools easier to access and use.

**MATERIALS AND METHODS:**

The SPARK platform integrates clinical, molecular, multiomic, imaging, and patient-reported data generated from PanCAN's research initiatives. The platform is built on a cloud-based infrastructure powered by Velsera. Cohort exploration and browser capabilities are built using Velsera ARIA, a specialized product for leveraging clinicogenomic data to build cohorts, query variant information, and drive downstream association analyses. Data science and analytic capabilities are also built into the platform allowing researchers to perform simple to complex analysis.

**RESULTS:**

Version 1 of the SPARK platform was released to pilot users, who represented diverse end users, including molecular biologists, clinicians, and bioinformaticians. Included in the pilot release of SPARK are deidentified clinical (including treatment and outcomes data), molecular, multiomic, and whole-slide pathology images for over 600 patients enrolled in PanCAN's Know Your Tumor molecular profiling service.

**CONCLUSION:**

The pilot release of the SPARK platform introduces qualified researchers to PanCAN real-world patient health data and analytical resources in a centralized location.

## Teaser

Access & use real-world #pancreaticcancer #healthdata through @PanCAN's SPARK cloud-based platform.

## INTRODUCTION

Pancreatic cancer is a lethal malignancy and holds the position of third deadliest cancer in the United States.^1^ Current therapeutic options offer a dismal overall survival (the 5-year survival is among the lowest for major cancers at approximately 12%^1^) and there are no widely adopted early detection methods for this disease. Thus, analysis of the clinical and molecular underpinnings of pancreatic cancer is critical to developing both early detection methodologies as well as novel therapeutic options. The Pancreatic Cancer Action Network (PanCAN) has been dedicated to fighting pancreatic cancer in a comprehensive manner for over 20 years. PanCAN provides direct support to patients, raises awareness, advocates for government support, and directly funds research through a competitive grants program and specialized clinical initiatives. PanCAN has a Patient Registry in which patient-reported information related to the pancreatic cancer patient experience is collected through deployment of surveys.^2^ PanCAN's Know Your Tumor (KYT) precision medicine service is provided free of charge to patients to perform molecular profiling of tumor tissue and report on personalized therapeutic and clinical trial options.^3^ Precision Promise (PrP) is PanCAN's multicenter, phase II/III adaptive clinical trial platform designed to evaluate and test multiple novel therapies compared with standard of care in patients with metastatic pancreatic cancer.^4^ PanCAN's Early Detection Initiative is a prospective clinical trial program designed to evaluate patients with new-onset hyperglycemia and diabetes through imaging and electronic medical records (EMRs) to retrospectively assess patients for early signs of pancreatic cancer.^5^ These combined PanCAN initiatives generate molecular, multiomic, clinical, patient-reported, treatment, outcomes, and imaging data. SPARK, PanCAN's health data integration platform, enables researchers to access, explore, analyze, and download (if needed) this rich resource of deidentified pancreatic cancer patient data.

**CONTEXT**

**Key Objective**
To introduce Pancreatic Cancer Action Network's (PanCAN) SPARK, a cloud-based data and analytics platform that integrates anonymized clinical, patient-reported outcome, multiomic, and imaging data from the PanCAN clinical and research initiatives.
**Knowledge Generated**
SPARK breaks down the traditional barriers to accelerating research by making real-world patient health data and analysis tools easier to access and use. Sharing data in this manner brings pancreatic cancer researchers closer to identifying novel treatments and ultimately improving patient outcomes.
**Relevance *(J.L. Warner)***
Platforms such as SPARK are an important step toward democratization of real-world data, with potential downstream patient benefits.*
  *Relevance section written by *JCO Clinical Cancer Informatics* Editor-in-Chief Jeremy L. Warner, MD, MS, FAMIA, FASCO.

Although several consortia have established large oncology data resources (eg, The Cancer Genome Atlas [TCGA],^6^ Clinical Proteomic Tumor Analysis Consortium [CPTAC],^7^ International Cancer Genome Consortium [ICGC],^8^ etc) along with complementary data repositories (eg, Genomic Data Commons [GDC],^9^ cBioPortal,^10^ etc) over the past decade, none have been dedicated solely to pancreatic cancer data. The aggressiveness and deadly nature of this disease warrants development of a data repository and analysis system dedicated to pancreatic cancer data. SPARK as a cloud-based data repository of pancreatic cancer data backed by an analysis engine will fill an unmet need in pancreatic cancer research. Rapid and efficient access to data in SPARK aligns with PanCAN's mission to accelerate research and foster collaboration in the field to improve patient outcomes.

## MATERIALS AND METHODS

### Data Generation, Sequencing, and Bioinformatics

DNA and RNA sequencing and bioinformatics analysis for KYT and PrP data in SPARK are conducted by Tempus since 2019. FASTQ files are aligned to hg19 genome build using the Novoalign alignment algorithm. Variants are called from the resulting alignment files using an analysis pipeline that detects single-nucleotide polymorphisms and indels using Freebayes and Pindel.^11,12^ Paired sample variant calling is performed on tumor samples and their respective matched normal controls, when available. Tempus' RNA assays are based on exome-capture RNA-Seq.

Tumor RNA .bam files are generated via STAR (version 2.5.4a) using the genomic reference, Ensembl GRCh37 Release 97. Transcript-level pseudoalignment and quantification to the Ensembl GRCh37 Release 97 (July 2019) reference is performed using kallisto (version 0.44). Solid tumor assays and whole blood are used to detect somatic and germline mutations, microsatellite instability, tumor mutation burden, chromosomal rearrangements, and copy-number alterations.^13-15^ For KYT clinical data, a Tempus algorithm is used to extract and assign line of therapy data for each patient from clinical documents and EMRs.^16^

### Data Normalization and Modeling

To ensure that KYT data from disparate sources have a unified representation, Tempus uses a normalization process consisting of standardized vocabularies that are commonly used in the industry to represent concepts as part of the data model. For example, LOINC is the target vocabulary used to represent laboratory test concepts, while RXNORM is the target vocabulary used to represent clinical drugs. Normalization is achieved through the use of mapping automation tools depending on the source, format, and domain of the concept, with additional quality control steps to ensure accuracy.

Within the SPARK platform, the data model is then reorganized in a user-friendly manner through the cohort browser user interface. Fields are grouped into main fields and property fields; main fields are those used for filtering or cohort definition and property fields are those that relate to and describe the main fields. The data are then grouped into categories on the basis of subject matter to facilitate a seamless cohort filtering user experience (demographics, laboratory tests, lines of therapy, etc)

Since the various PanCAN data assets are generated from disparate data sources each with aims to capture diverse data types, there is not a common data model across all of the data assets. However, the above-described data model framework can be applied to both current and future data generated through PanCAN's research initiatives.

### Cloud Platform/System Architecture

SPARK is built on the Velsera Seven Bridges Core Infrastructure (SB-CI)—a system of more than 50 microservices that support the entire spectrum of biomedical data and analysis. SB-CI's cloud-agnostic–multicloud capability allows researchers to connect and send analyses to data located in another cloud provider or region, for example, PanCAN data in Amazon Web Services (AWS) or healthy controls in Google Cloud Platform.

### Interactive Cohort Exploration and Data Analysis

**FIG 1.**
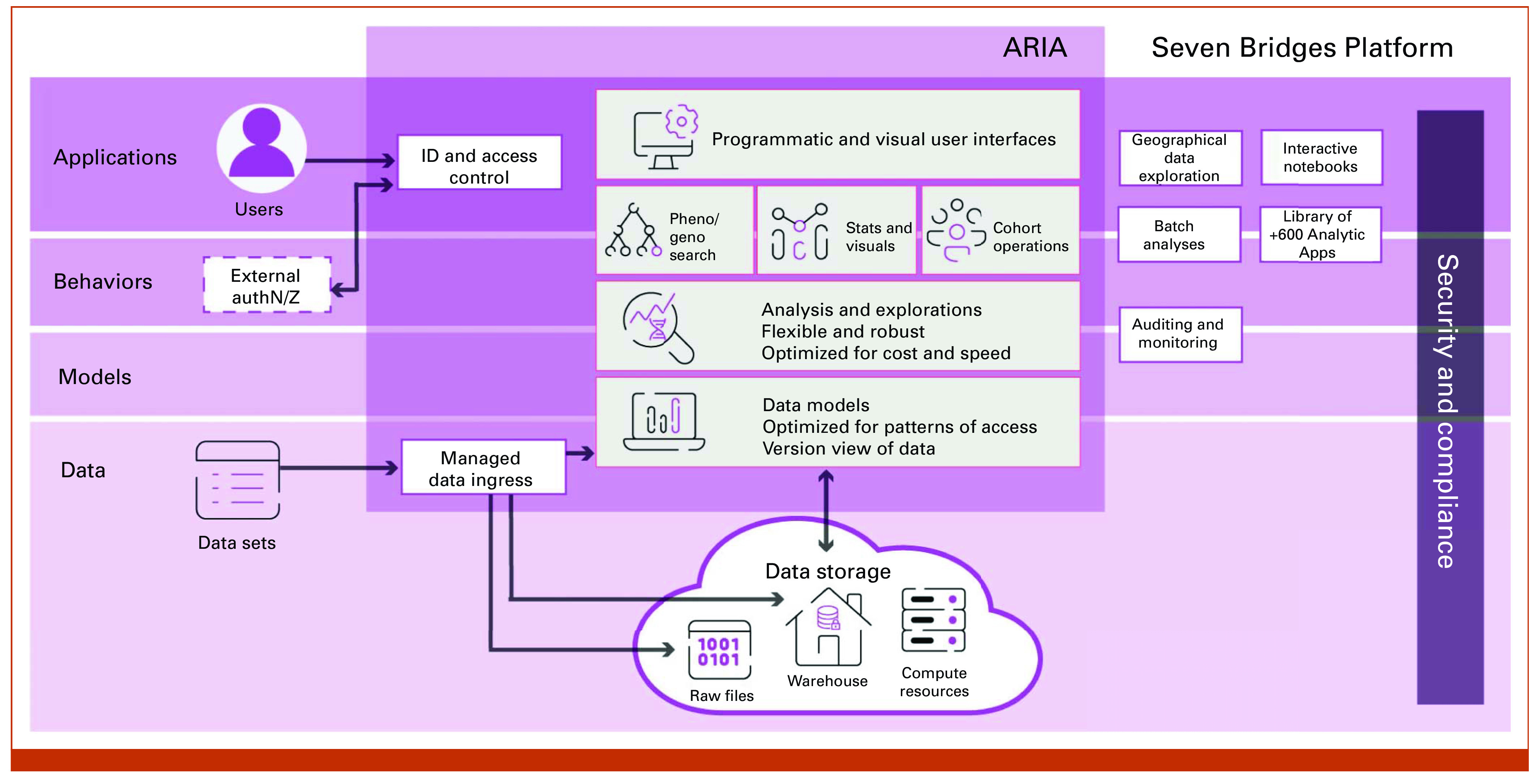
The PanCAN SPARK platform is built on the Velsera SB-CI. ARIA, a specialized product for leveraging clinicogenomic data to build cohorts on clinical, demographic, and molecular data (including genomic and transcriptomic data) and query variant information, layers on top of the SB-CI to create an end-to-end user experience. ID, user identification; PanCAN, Pancreatic Cancer Action Network; SB-CI, Seven Bridges Core Infrastructure.

The SPARK cohort browser allows users to explore and build cohorts on clinical, demographic, and molecular (including genomic and transcriptomic) data. The cohort browser is built on Velsera Seven Bridges ARIA, a specialized product for leveraging clinicogenomic data to build cohorts, query variant information, and drive downstream association analyses (Fig 1). The data sets can be explored either by filtering on the data fields or by filtering on metadata associated with the raw files (Data Supplement, Video S1). Analyses can be conducted within the project space on the platform where users export results from queries. The platform has over 700 pipelines/workflows available for raw multiomics data analysis. Data can also be explored with the interactive Data Studio using either Jupyter Notebooks or RStudio on customizable cloud-based virtual machine instances.

Users have the ability to copy tools, pipelines, or workflows to their projects to conduct analyses. Once copied, tools, pipelines, and workflows can be executed by creating tasks which encompass all details of an execution, including the tool/pipeline/workflow and associated version, input or reference files used, parameters used, outputs, and operational details such as date/time analysis was executed, cloud costs, as well as detailed logs. Tasks cannot be deleted or modified once they have been initiated, ensuring that analyses on the SPARK platform are fully reproducible and traceable. Data provenance is also ensured as the files and data generated by tasks are linked to the specific task that created them. In this way, the platform ensures that all execution details in addition to input and output files can be traced.

### Data Sharing and Access

All patient data in SPARK are deidentified and hosted on the platform for research and educational purposes only. We work in concert with regulators, institutional review boards, and our partners to ensure that the data released to researchers are done so in alignment with patient consents, regulatory guidelines, and all applicable laws.

To safeguard patient data, interested researchers and clinicians (also including peer reviewers) will be required to submit a PanCAN data use agreement (DUA) to gain access to the SPARK platform (DUA can be found at PanCAN SPARK^17^). Upon submission of the DUA, we strive to grant access to qualified researchers within 48 hours.

### Advisory Committee

An Advisory Committee consisting of leaders in pancreatic cancer research and/or data science from academia and industry was assembled to ensure development of the platform continues to meet the needs of the pancreatic cancer research community.

## RESULTS

Version 1 of the SPARK platform was released to pilot users, who represented diverse end users, including molecular biologists, clinicians, and bioinformaticians (homepage: PanCAN SPARK^17^). The landing page guides prospective and returning researchers on portal access. New users can request access by submitting the DUA while returning users are prompted to enter their login credentials.

**FIG 2.**
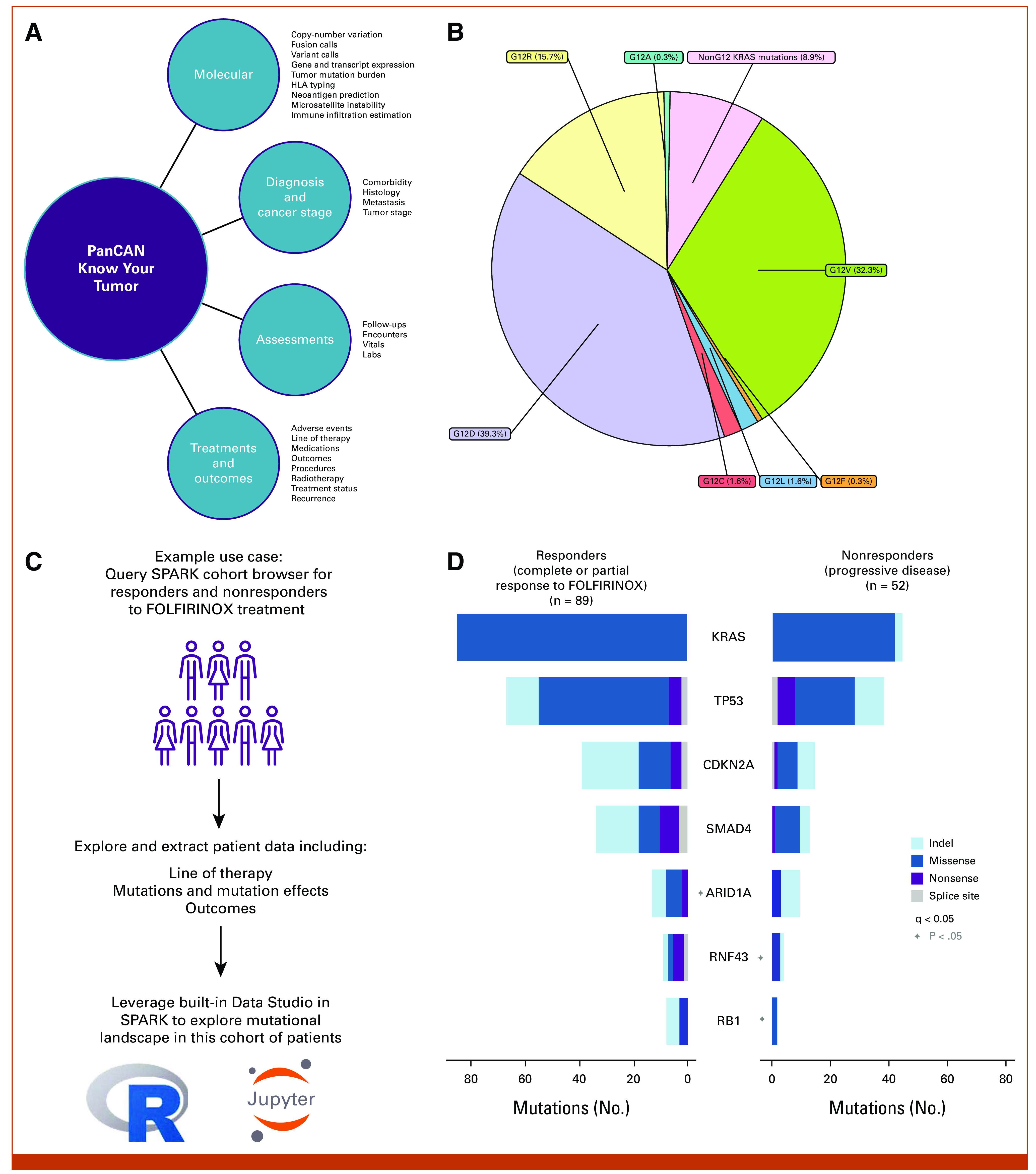
(A) KYT data can currently be explored in SPARK by filtering on multiple molecular and clinical features. (B) Distribution of G12/non-G12 KRAS mutations across all PDAC patients in the KYT data set. (C) Exploratory data analysis can be conducted using powerful and collaborative interactive data analytics capabilities in the built-in Data Studio. (D) Driver gene discovery analysis was conducted in RStudio using the dNdScv algorithm with default parameters. The mutational patterns of the significantly mutated genes for responders and nonresponders for KYT patients. KYT, Know Your Tumor; PanCAN, Pancreatic Cancer Action Network; PDAC, Pancreatic Ductal Adenocarcinoma.

Included in the pilot release of SPARK are deidentified clinical, molecular, imaging, treatment, and outcome data for over 600 patients enrolled in PanCAN's KYT program (Fig 2A). Additional KYT patient data are added/refreshed quarterly. The platform will ultimately serve as a consolidated repository for all of PanCAN's patient health data (Table 1). Integration of public pancreatic cancer data from sources such as TCGA or CPTAC is enabled through Velsera's Seven Bridges Cancer Genomics Cloud^18^ allowing researchers to compare public data sets with data from PanCAN-sponsored studies.

**TABLE 1.**
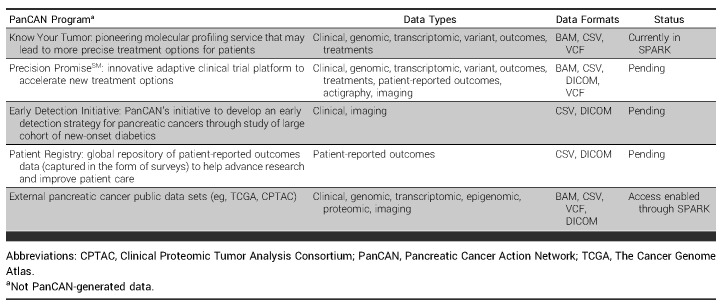
PanCAN's Initiatives That Generate Patient Health Data

At present, KYT cohort data can be explored by filtering on multiple molecular and clinical fields, including demographics, cancer stage, laboratory tests, and treatments and outcomes data (Data Supplement, Video S1). Graphical visualizations and tabular representations of the results are returned. Users can then bring these results into their projects (workspaces) for statistical analysis, prototyping algorithms, and visualizations via interactive analysis. Alternatively, these data can also be downloaded for use in external analytics platforms.

### Use Case Example

The initial KYT data on the platform are heterogeneous in nature and were derived from multiple tissue sites across multiple cancer stages as well as diverse tumor histologies, allowing for an in-depth analysis of pancreatic cancer. KYT data can currently be explored through the SPARK cohort browser by filtering on multiple molecular and clinical features (Fig 2A). The use-case examples in Figures 2B-2D demonstrate how various data types (treatment, clinical, and variant lesion data) can be integrated and analyzed in conjunction within the platform. For example, it is possible to conduct a survey of the KRAS G12 mutations among KYT patients (Fig 2B). As an illustration of the utility, functionality, and available data in the SPARK platform, we compared somatic mutation patterns between responders and nonresponders to FOLFIRINOX, a first-line standard-of-care treatment for pancreatic cancer. Starting with the cohort explorer, we queried for somatic mutations and mutation effects data in KYT patients with pancreatic cancer who had a complete or partial response (responders) as well as those who had no response (nonresponders) to FOLFIRINOX treatment (Fig 2C). Variant data for these cohorts were parsed and explored using the built-in RStudio implementation. Driver gene discovery analysis was conducted in RStudio using the dNdScv algorithm with default parameters.^19^ The mutational patterns of the significantly mutated genes for these patients are presented in Figure 2D. Data derived from these types of analyses can be further integrated with other available clinical data such as CA 19-9 levels or outcomes data as well as with other molecular data (eg, gene expression data) in SPARK. These data can also be compared with known mutational frequencies such as from the TCGA PAAD data set (eg, KRAS 86% in KYT to 90.7% in TCGA; Data Supplement, Table S1). With both rich patient data sets as well as analytical capabilities, the platform affords the opportunity to seamlessly conduct data discovery, exploration, and analysis in a central location.

## DISCUSSION

Landmark multiomics studies such as TCGA generated petabytes of data and enabled researchers to study cancer at multiple levels of biological organization (ie, genomic, transcriptomic, and epigenomic). Initiatives such as National Cancer Institute's Cancer Research Data Commons (CRDC) paved the way for democratizing access to publicly available cancer data by breaking down data silos and ensuring qualified researchers can access and analyze data in centralized repositories. Using the model of the CRDC, we present here SPARK, a cloud-based platform that serves to integrate PanCAN's deidentified patient health data (including clinical, imaging, molecular, treatment, and patient-reported outcomes) and allows qualified researchers open access to rich pancreatic cancer data sets. Design and development of the SPARK platform was guided by a group of representative end users, including over 80 researchers and key leaders in the pancreatic cancer research community.

SPARK is still under development with continuous improvements, new features as well as new data being added on the basis of feedback from pilot users and the pancreatic cancer research community. The pilot release introduces qualified researchers to the data resulting from PanCAN's KYT program and the analytical capabilities of the platform. With the data and analytical tools in a centralized location, SPARK brings pancreatic cancer researchers closer to identifying novel treatments and ultimately improved patient outcomes.

## Data Availability

All patient data in SPARK are deidentified and hosted on the platform for research and educational purposes only. We work in concert with regulators, institutional review boards, and our partners to ensure that the data released to researchers is done so in alignment with patient consents, regulatory guidelines, and all applicable laws. To safeguard patient data, interested researchers and clinicians (also including peer reviewers) will be required to submit a Pancreatic Cancer Action Network data use agreement (DUA) to gain access to the SPARK platform (DUA can be found at www.pancan.org/spark). Upon submission of the DUA, we strive to grant access to qualified researchers within 48 hours.
